# Stimuli-Responsive Polymeric Systems for Controlled Protein and Peptide Delivery: Future Implications for Ocular Delivery

**DOI:** 10.3390/molecules21081002

**Published:** 2016-07-30

**Authors:** Pakama Mahlumba, Yahya E. Choonara, Pradeep Kumar, Lisa C. du Toit, Viness Pillay

**Affiliations:** Wits Advanced Drug Delivery Platform Research Unit, Department of Pharmacy and Pharmacology, School of Therapeutic Science, Faculty of Health Sciences, University of the Witwatersrand, Johannesburg, 7 York Road, Parktown 2193, South Africa; pakama.mahlumba@students.wits.ac.za (P.M.); yahya.choonara@wits.ac.za (Y.E.C.); pradeep.kumar@wits.ac.za (P.K.); lisa.dutoit@wits.ac.za (L.C.T.)

**Keywords:** bioavailability, in situ, ocular barriers, ocular delivery, pre-corneal elimination, protein and peptide delivery, stimuli responsive polymer

## Abstract

Therapeutic proteins and peptides have become notable in the drug delivery arena for their compatibility with the human body as well as their high potency. However, their biocompatibility and high potency does not negate the existence of challenges resulting from physicochemical properties of proteins and peptides, including large size, short half-life, capability to provoke immune responses and susceptibility to degradation. Various delivery routes and delivery systems have been utilized to improve bioavailability, patient acceptability and reduce biodegradation. The ocular route remains of great interest, particularly for responsive delivery of macromolecules due to the anatomy and physiology of the eye that makes it a sensitive and complex environment. Research in this field is slowly gaining attention as this could be the breakthrough in ocular drug delivery of macromolecules. This work reviews stimuli-responsive polymeric delivery systems, their use in the delivery of therapeutic proteins and peptides as well as examples of proteins and peptides used in the treatment of ocular disorders. Stimuli reviewed include pH, temperature, enzymes, light, ultrasound and magnetic field. In addition, it discusses the current progress in responsive ocular drug delivery. Furthermore, it explores future prospects in the use of stimuli-responsive polymers for ocular delivery of proteins and peptides. Stimuli-responsive polymers offer great potential in improving the delivery of ocular therapeutics, therefore there is a need to consider them in order to guarantee a local, sustained and ideal delivery of ocular proteins and peptides, evading tissue invasion and systemic side-effects.

## 1. Introduction

Therapeutic proteins and peptides are advantageous over small molecule drugs in that they mimic similar molecules found in the human body; they are biocompatible and highly potent. However, limitations (both drug related and patient related) do exist, caused by their high molecular weight, poor transfer across biological membranes, provocation of immune responses, short half-lives and instability of the molecules [1,2]. The controlled release of therapeutic proteins is regarded as a way to increase the efficacy while reducing side effects, and therefore improving the patient’s quality of life [3].

The most commonly used route of administration for proteins and peptides is the parenteral route, i.e., injection/intravenous infusion providing direct administration into the bloodstream. Though this route can overcome some of the limitations, it is costly, painful and results in low patient acceptance [4]. Alternative routes comprise of the nasal route which provides rapid absorption of the active agent, increased bioavailability and ease of administration, and bypasses gastrointestinal tract-related degradation. The use of nanoparticles as a carrier allows delivery directly into the brain via the nasal route but the dose range is restricted by the surface area of the nasal epithelium. The oral route has come across a number of challenges involving probable degradation ascribed to the acidic environment in the stomach, first pass metabolism in the liver and the proteolytic enzymes in the intestinal tract. The pulmonary route with its large surface area and the thin alveolar epithelium permits rapid protein/peptide absorption and also avoids first pass metabolism. The vaginal route can be used for systemic delivery of proteins and peptides through the rich blood supply in the vagina. Numerous limitations exist in this route as it is specific for females, sensitive, associated with irritation and patients are reluctant to use this route. The transdermal route favours the delivery of proteins and peptides with short half-lives and provides sustained delivery. The limitation with this route is the stratum corneum which has a low permeability and this becomes a major challenge in the delivery of proteins and peptides due to their size, therefore modifications of the drug delivery systems is required.

Much attention is drawn to the ocular route for the reason that this review focuses on the future prospects in responsive ocular delivery of macromolecules. The ocular route is potentially useful for systemic delivery of therapeutic proteins and peptides; however it is often used for localized delivery of ophthalmic agents. It is the most interesting and challenging route in drug delivery due to the sensitive and complex environment of the eye [5]. The eye is a globular sensory structure encasing the optical framework in a three layer membrane that isolates it from the systemic circulation [6]. The outermost layer responsible for mechanical support is the protective fibrous sclera with its anterior portion transparent to visible light, called the cornea. The middle layer is formed by a network of capillaries called the choroid which regulates circulation in and out of the retina. The innermost layer is the photosensitive retina with photoreceptor cells which process light information, convert it to electrical impulses sent to the brain via the optic nerve and interpreted as vision [6,7].

The ocular route can further be divided into the topical, intraocular and periocular routes of drug delivery. The topical route can be corneal, in the order of passage through the cornea→aqueous humour→intraocular tissue, or non-corneal, via conjunctiva→sclera→choroid or RPE. It is the easiest route for delivery of small molecules to the anterior segment, however, it is inadequate for the posterior segment. Less than 5% of the instilled dose enters the eye and only 0.001% is predicted to reach the posterior segment; macromolecules cannot cross the cornea. This low bioavailabity is due to the short retention time of the instilled drugs in the ocular surface, limited volume that can be administered (~30 μL), as well as the corneal epithelium barrier [8]. The cornea forms a significant barrier in the anterior segment for topical drug administration while the sclera in the posterior is fairly permeable. The blood-retinal barrier (BRB) and the neural retina are rate-limiting factors in the passage and absorption of materials, particularly macromolecules due to large size, from the choroid to the retina and vitreous in the posterior segment which reduces bioavailability (1%–2%) in systemic delivery.

The neural retina is a multilayered membrane (inner and outer limiting membranes) separating pigmented retinal layer and the vitreous humour. The BRB is made of tight junctions of retinal endothelial blood vessels and retinal pigment epithelium (RPE) [9]. These are factors that need to be considered in ocular delivery of macromolecules. Intraocular delivery includes intravitreal, suprachoroidal, intrastromal, intracameral, intrascleral and subretinal routes. The intraocular route deposits drug directly in the eye (or at the target site). This route bypasses tissue barriers, bioavailability is increased and side effects reduced due to targeted delivery. The periocular route is the delivery of active agents into the region encircling the eye; it comprises of the subtenon, peribulbar, posterior juxtascleral, retrobulbar and subconjunctival injections. Though this route is not as effective as intraocular injections, it is less invasive, with better bioavailability compared to the topical route. In addition, significantly larger volumes of drug solution (500–5000 μL) can be contained in periocular delivery compared to intravitreally administered volume (50–100 μL) [9,10].

A number of factors pose a challenge in ocular delivery of therapeutic proteins and peptides. These factors include decreased penetration of proteins and peptides into target sites, leading to insufficient levels at that target site, limited diffusion into ocular tissue due to large size, further making topical delivery almost impossible, reduced bioavailability caused by decreased residence time, invasive and frequent administration leading to poor patient compliance, and instability of these molecules. Additionally, the small size of the eye along with the challenges faced prove there is a need for targeted delivery in order to improve the intended therapy [11,12,13]. A group of polymers referred to as stimuli-responsive materials is currently being investigation for use in the delivery of therapeutic proteins and peptides.

Stimuli-responsive polymers (Table 1) are defined as materials that display rapid physicochemical transitions in response to small changes in the surrounding environment [14]. Such polymers have become part of the main focus in biomedical applications and drug delivery platforms due to their distinct features and ability to control drug release [15]. The classification of these polymers is based on two controls, the proximal (disease specific) and the remote (non-disease specific) control of stimuli responses, further classified as physical or biochemical stimuli. Desirable characteristics include biodegradability, biocompatibility, contaminant/pyrogen-free, non-toxic, low cost, high loading capacity and an excellent stability profile. The use of these polymers provides a less invasive delivery approach via externally controlled stimuli and extended stability in the body owing to their stimuli specificity and therefore site-specific release [14]. Their reversible mechanism upon removal of the trigger is likely to minimize the possibility of drug-related toxicity. Stimuli-responsive polymeric delivery systems have a potential to at most overcome the challenges faced with ocular protein and peptide delivery. A group of stimuli-responsive drug delivery systems known as in situ forming systems has increasingly gained interest over the years. These systems consist of phase transition polymers which enable them to be injectable solidifying at the site of delivery via response to various stimuli such as ions, pH and temperature. In situ formation can occur as a result of physical or chemical alteration in the delivery system. The rationale for these systems arises from a number of formulation shortcomings which will be discussed in detail below [16,17].

This review explores possible alternatives for responsive ocular protein/peptide delivery by evaluating various stimuli employed, responsive systems that have been designed to deliver these molecules as well as therapeutic proteins and peptides used in the treatment of ocular disorders. Examples of stimuli included are pH, temperature, glucose, enzymes, light, ultrasound and magnetic field. This work also considers advances in stimuli-responsive ocular delivery systems that have been designed over the years. This work evaluates the future of stimuli-responsive delivery systems in ocular delivery of therapeutic proteins and peptides.

## 2. Responsive Protein and Peptide Delivery

Proteins and peptides are widely used in the field of therapeutics mainly because of their ability to mimic those that are produced by the human body, which renders them compatible with the body and confers them with relatively high potency. Biodegradable polymers have been used to achieve a delivery strategy that protects proteins and peptides from the human body and vice versa. These polymers are able to encapsulate proteins and peptides and release following degradation kinetics; however, when other formulation factors dominate they may interfere with degradation kinetics [29]. Using degradable responsive polymers and attaching responsive moieties to the polymers in use gives rise to stimuli-responsive polymeric systems which are capable of sensing surrounding environmental changes and in response release the encapsulated therapeutic protein or peptide [14,30]. Stimuli-responsive polymers have the ability to respond to stimuli present in the human body enabling site specific delivery of therapeutic proteins and peptides. Response to stimulus can occur via formation or destruction of secondary forces such as hydrogen bonding and simple reactions like acid-base reaction from the responsive moieties in the polymer [15]. These are advantageous in that they are capable of on and off release, that is, only the amount of drug required for a therapeutic effect at the time is released. Table 2 summarizes various delivery systems that employ responsive delivery of proteins and peptides.

### 2.1. pH-Responsive Systems

The most used stimulus in oral drug delivery of proteins and peptides is pH. This is to avoid the degradation of these agents, however the stimulus can be employed ocularly, especially for topical delivery, to increase residence time via targeting the pH of the tear fluid. Ducat and coworkers [31] proved the benefit of using pH-sensitive liposomes in preference to conventional liposomes as a vector for the delivery of a peptide that antagonizes Print3G, a breast cancer oncoprotein, the rationale being that the pH-responsive liposomes can improve the efficacy and site specificity as they release the peptide inside the cell. The mechanism of these liposomes lies in the presence of the compound cholesteryl hemisuccinate (CHEMS), which possesses an inverted cone shape at physiological pH. Under acidic conditions, the carboxylic acid group becomes protonated and CHEMS loses this shape releasing the protein. This transition occurs within the endosome where pH is decreased during endocytosis [32]. The endosomal release of encapsulated Print3G observed with pH sensitive liposomes allows for site specific release. The results obtained in this study proved pH-sensitive liposomes to be beneficial and efficient over classical liposomes in intracellular delivery of peptides. In another study, Koyamatsu and colleagues [3] designed reverse polymer micelles using biodegradable, biocompatible poly(ethylene glycol) (PEG) and poly (d,l-lactic-*co*-glycolide) (PLGA) with the terminal carboxyl group in PLGA conferring the pH responsiveness of the micelles. Bovine serum albumin (BSA) and streptavidin were used as model proteins to prove the encapsulation and controlled release of large molecules from the micelles. The mechanism of release is based on the ability of the carboxyl group to undergo deprotonation at physiological pH, thus compromising the hydrophobic layer and releasing the protein into the target site. A low cytotoxicity micelle-based protein drug carrier with pH-dependent targeted release was achieved and encapsulation of macromolecules was successful [33]. The swelling behavior of hydrogels in combination with pH sensitivity is observed in microspheres and beads designed by El-Sherbiny and Gong et al., respectively. In vitro results showed sustained release of BSA (model protein) from the microspheres, highest entrapment capacity >80% proving the capability to encapsulate macromolecules. These hydrogel based systems are designed to swell at designated pH and release the loaded protein bypassing degradants, localizing therapy, therefore improving therapeutic outcomes and bioavailability [34,35].

### 2.2. Thermo-Responsive Systems

Temperature as a stimulus can either be external, that is change in temperature due to an external trigger, e.g., radiation, or internal, which is change in temperature caused by the disease state such as inflammation. These changes in temperature can be used to trigger drug delivery at higher or lower temperatures, depending on the nature of the polymer employed. Thermo-responsive polymers have a lower critical solution temperature (LCST) which is the highest temperature at which the polymer is soluble and above that temperature they become insoluble or upper critical solution temperature (UCST), the lowest temperature of miscibility [42,43]. Hydrogels are mostly preferred because of their macroporous structure and good swelling properties. In the delivery of proteins and peptides, their macroporous structure makes them good and efficient carriers for such macromolecular drugs. Responsive hydrogels are especially good for sustained delivery of proteins and peptides as their aqueous environment is able to protect these fragile drugs [44]. An example of investigated responsive delivery of proteins is a poly(*N*-isopolyacrylamide) (NIPAM)-based implantable hydrogel designed by Ninawe and colleagues [45]. In this study NIPAM with LCST of 32 °C was used as the temperature responsive gelling polymer and a model protein immunoglobulin G (IgG) was loaded into the gel for delivery to the posterior segment of the eye via implantation in the sclera as the treatment of age-related macular degeneration (AMD). After implantation, the gel uses the deswelling mechanism when it reaches body temperature releasing the drug to diffuse across the sclera reaching the target site which is the choroid and RPE. When deswelling stops, the remaining (30%) protein is released via diffusion over a minimum period of 25 h. The study predicts a less frequent dose administration of at least two month intervals with the hydrogel compared to intravitreal injection. Thermo-responsive systems can also enhance selectivity for target tissue to protect normal tissue from damage by the therapeutic peptides.

### 2.3. Enzyme-Responsive Systems

Enzymes are biocompatible, function under mild conditions, and display a high degree of selectivity and these are advantages that make them good candidates as triggers for stimuli- responsive release. These can be enzymes responsible for degradation or enzymes over-expressed in disease states (disease-specific). Upon exposure to the target enzyme (thermolysin), selective enzymatic hydrolysis of the enzyme-cleavable peptide resulted in the release of anionic fragments, leaving anchored cationic fragments that convert the neutral poly(ethylene glycol acrylamide) particles into cationic particles. This charge switch leads to particle swelling and a significant increase in overall particle diameter, a visual indication of increased internal mesh size. This allows the triggered release of entrapped proteins pre-loaded into the particles [46]. Aimetti and co-workers [47] report on a study of enzyme-responsive hydrogel using human neutrophil elastase (HNE), secreted by neutrophils present at the inflammation site, as the triggering enzyme for the hydrogel in the delivery of anti-inflammatory therapeutics. The hydrogel only releases at the site of inflammation due to the presence of neutrophils and performs localized and site specific delivery avoiding drug-related systemic side-effects. HNE-sensitive linkers were added to poly(ethylene glycol) diacrylate (PEGDA) for the sensitivity of the hydrogel and model peptides were incorporated. HNE diffuses into the hydrogel and splits the substrate releasing the therapeutic agent at the site of action. Localized release of the peptides in the presence of HNE was achieved in vitro. In another study done by Itoh and co-workers [48], enzymatic degradation which is experienced with most drug delivery systems in the body was used as an aid to achieve controlled delivery of protein drugs. Fluorescein isothiocyanate-labeled albumin (FITC-albumin) was used as the model protein to study drug loading and release behaviour of the hollow capsules that were designed. Enzymatic degradation by chitosanase weakens the capsule wall causing it to wrinkle and change shape releasing the therapeutic protein. No change in the capsule was observed in the absence of chitosanase therefore the degradation is specific to the enzyme chitosanase and the capsule is biodegradable, destroyed over time. The cationic chitosanase is adsorbed in larger amounts on anionic surface leading to faster enzymatic degradation of the capsule wall compared to adsorption observed on cationic surface. The amount released from positively charged capsules was 28% within 24 h and 50% in 4 days while negatively charged capsules released 55% in 24 h and 63% in 4 days. These results show that the rate of release from the hollow capsules can be manipulated by altering the surface charge of the capsule to achieve prolonged release.

### 2.4. Light-Responsive Systems

**Figure 1:**
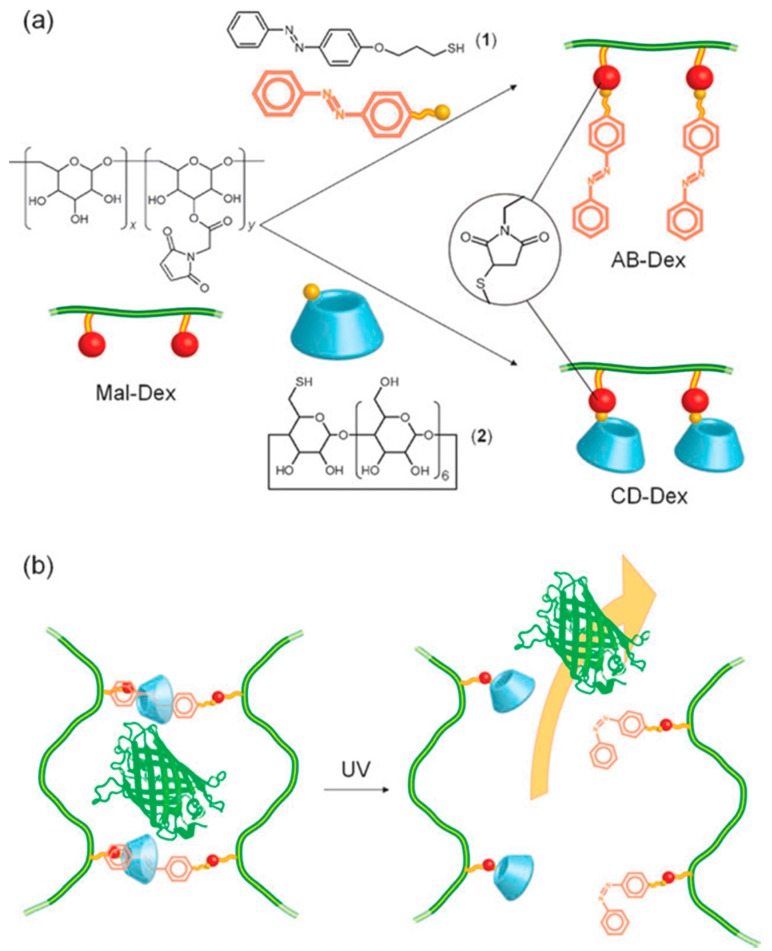
(**a**) Preparation of azobenzene-modified dextran (AB–Dex) and cyclodextrin-modified dextran (CD-Dex) through the thiol–maleimide reaction; (**b**) Schematic representation of photoresponsive protein release from the gel composed of trans AB–Dex and CD–Dex. Upon UV light irradiation azobenzene moieties isomerise from *trans* to *cis* configurations, resulting in the dissociation of crosslinking points, and allowing the entrapped protein to migrate into the media [51]. (Reproduced from Peng et al. [51] with permission of The Royal Society of Chemistry.)

Amongst the stimuli employed in drug delivery, light has been found to be one of the more interesting stimuli due to its remote action as a stimulus allowing spatial control and convenience [49]. Photoresponsiveness is exhibited through photochromic photoswitchable molecules such as spyropyran, anthracene and azobenzene which make the system phototriggerable [50]. Figure 1 shows azobenzene-modified dextran and the mechanism of phototriggered protein release.

Kang and co-workers [39] reported on light responsive core-shell nanogels formed from gold-silver nanorods (Au–Ag NRs) coated with polymeric shells cross-linked with DNA. Doxorubicin was used as a model drug encapsulated in the gel scaffold. Au-Ag NRs act as photothermal convectors by absorbing near infrared photon energy which is then converted to heat and elevates temperature at the target site therefore increasing membrane permeability. Nanogels were irradiated with a near-infrared (NIR) laser beam (808 nm) and a burst release of 65% ± 7% was observed within 10 min. Results from this work confirm the potential of the nanogels in remote controlled targeted delivery to obtain optimal drug levels in targeted tumor cells [52,53]. Light responsive nanosized polyion complex micelles with switchable surface charge designed by Jin et al. [54] are nanovehicles suitable for intracellular delivery of proteins. The polyion complex is formed by a light responsive block copolymer, poly (*N*,*N*-dimethyl-*N*-(2-(methacryloyloxy)-ethyl)-*N*-((2-nitrobenzyl)oxy)-2-oxoithaminium bromide)-block (carboxybetaine methacrylate) (PDMNBMA-b-PCBMA) with BSA through electrostatic interaction. The surface charge of the polyion complex is changed at the tumor site (acidic pH) from neutral (physiological pH) to positive due to carboxylate group protonation. UV irradiation causes transformation of positively charged PDMNBMA blocks to zwitterionic carboxybetaine which results in polyionic complex micelles disassembling and BSA being released promptly. In the absence of UV irradiation ~20% of BSA was released and 77.3% under UV irradiation both in 24 h, thus proving the photoresponsiveness of the micelles. This exogenously controlled light responsive delivery system was designed to overcome the controlled release challenge posed by physiological factors which are observed in the use of endogenous stimuli such as pH and temperature [55].

### 2.5. Ultrasound-Responsive Systems

Ultrasound is also an exogenous stimuli used as a trigger for drug release which employs pressure waves with frequency of about 20 kHz and above. The mechanism of ultrasound involves a medium that focuses and reflects and refracts the ultrasonic waves. Ultrasound-responsive drug delivery systems that have been designed are microbubbles, nanodroplets, nanobubbles, micelles, liposomes, etc. [56,57]. Wang and coworkers [58] investigated the ultrasound-responsive behavior of polymeric micelles formed from poly(ethylene oxide)-block-poly(2-tetrahydropyranyl methacrylate) (PEO-b-PTHPMA) an amphiphilic diblock copolymer. Upon exposure to high intensity focused ultrasound (HIFU), ultrasonic cavitation occurs at the site of action generating very high local temperature (500 K) and pressure (500 atm) leading to rapture of polymeric chains therefore releasing the encapsulated active agent [59].

### 2.6. Multi-Responsive Systems

**Figure 2:**
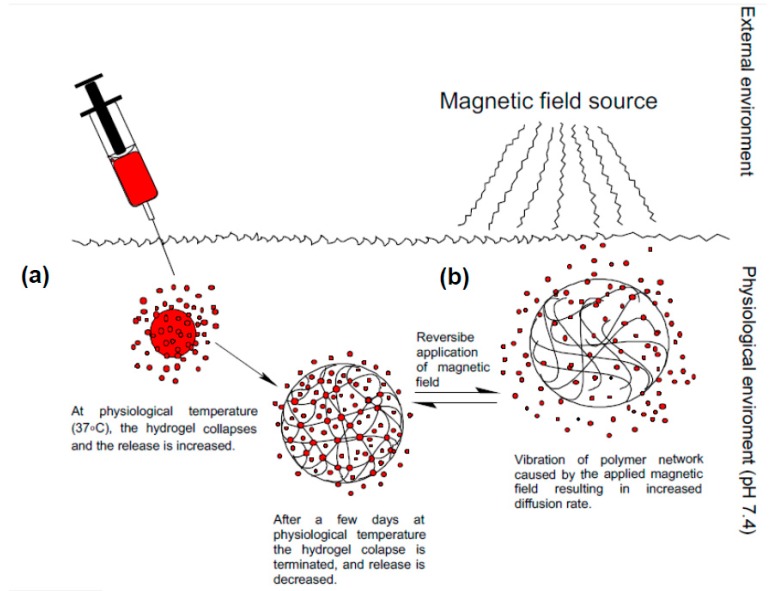
Illustration of the mechanism of drug release from a multi-stimuli responsive doxorubicin loaded hydrogel. (**a**) Physiological temperature (37 °C) causes the hydrogel to collapse and release doxorubicin; with time it regains its original state and (**b**) shows the application of external magnetic field causing the polymer network to vibrate and release doxorubicin via increased diffusion rate.

Multi-stimuli responsive drug delivery systems enhance sustained delivery by synergistically acting on the system resulting in a more improved control of release. These can involve a combination of materials that are responsive to two or more stimuli. An example is a multi-stimuli responsive hydrogel designed by Casolaro and coworkers [60] that included pH, temperature and magnetic field as stimuli. Casolaro used α-amino acid residues, including l-valine, structurally similar to poly (*N*-isopropylacrylamide) (pNIP) to form vinyl polyectrolyte hydrogels. The external stimulation of the magnetic field, through the magnetic nanoparticles embedded into the hydrogel, directs the system to the specific target tissue for localized release and also induces local hyperthermia stimulating the thermo-responsive part of the system. The mechanism of protein release from the hydrogel is illustrated in Figure 2. At pH 7.4 chain complexation occurs enhancing the stability of the polymer chains. At 37 °C, the hydrogel collapses and the release is increased until the collapse is terminated after a few days and the release is decreased. After this, the magnetic field is applied which causes vibration of the hydrogel network accelerating diffusion rate. The magnetic field is suspected to increase hydrogel temperature stimulating the thermo-responsive NIP. A sustained release of the active drug >25 days was observed in the multi-responsive hydrogel. The release rate can be manipulated remotely through external triggers, either by increasing the magnetic field strength.

Yin and coworker [61] prepared dual-responsive concanavalin A (Con A)-based microhydrogels sensitive to both pH and glucose for the delivery of insulin. The sensitivity of insulin release from microhydrogels was observed upon small changes in pH value, whereby insulin release was rapidly increased with a decrease in pH via swelling of the system due to ionization of the amino groups. Increasing the pH leads to hydrogen bonding within the amino groups which forms a complex that restricts the motion in polymer network chains and therefore restricts the release of insulin. Free glucose seizes the specific binding sites of Con A–polymer complex leading to the dissociation of the complex and resulting in a glucose sensitive delivery system. Results observed from characterization of the system show an increase in insulin release with increasing glucose concentration, also a rapid change was observed with every change in glucose concentration. The presence of glucose reduces crosslinking density in microhydrogels and increases their hydrophilicity resulting in swelling. The microhydrogels are biocompatible and make good systems for self-regulated delivery of insulin [46].

**Figure 3:**
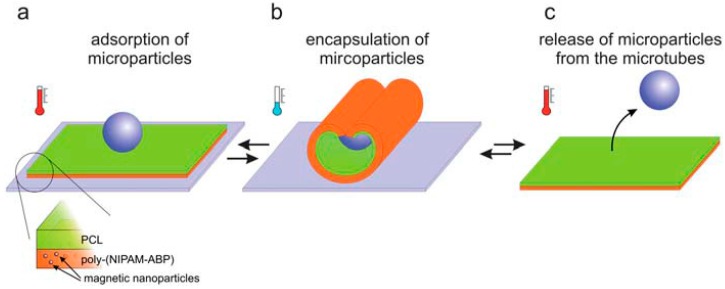
Schematic representation of the mechanism of encapsulation and release of microparticles by the bilayer self-folding microtubes. The microtubes are formed from a film of poly(*N*-isopropylacrylamide-*co*-4-acryloylbenzophenone) (poly(NIPAM-ABP) and polycaprolactone (PCL) mixed with magnetic nanoparticles. The microparticle (**a**) adsorbs into the bilayer film; at low temperature (less than LCST); the film (**b**) folds forming a microtube that encapsulates the microparticle; and upon high temperature (above LCST) (**c**) it unfolds releasing the microparticle. (Reproduced from Zakharchenko et al. [62] with permission of The Royal Society of Chemistry.)

Zakharchenko and colleagues [62] reported thermo-magneto-responsive bilayer self-folding microtubes as carriers of drug-loaded microparticles. The bilayer was made from a poly(*N*-isopropylacrylamide-*co*-4-acryloylbenzophenone) (poly(NIPAM-ABP)) copolymer which is a combination of thermoresponsive poly(*N*-isopropylacrylamide) (PNIPAM) and photoresponsive photocrosslinker, 4-acryloylbenzophenone (ABP) with hydrophobic polycaprolactone (PCL). PCL forms the outer layer of the microtube mixed with magnetic nanoparticles. At low temperature below its LCST (28 °C), the bilayer film folds and encapsulates the drug-loaded microparticles and at elevated temperature >28 °C, it unfolds and releases them. The mechanism of thermo-responsiveness of the microtubes is illustrated in Figure 3. Magnetic nanoparticles enable remote control of the microtubes via magnetic fields, which makes the system multi-stimuli responsive.

Another dual responsive delivery system designed by Garbern and coworkers [38] possessed responsiveness to pH and temperature. At higher temperatures, the hydrogel was soluble and formed a stable gel at body temperature. Decreasing the pH to ~5.5 resulted in gel formation. At pH 7.4, a rapid release of the protein from the hydrogel was observed and on the contrary at pH values between 5 and 6, the release was slowed over 28 days. This system was proved as providing sustained release of large molecules and allowing slow degradation of the polymer. Site specificity was not mentioned in the study done, however tuning of the delivery system gives a possibility of site-specific release and optimal bioavailability. The eye offers controls such as temperature and pH which pose as triggering stimuli for drug release; therefore the responsive delivery approaches mentioned above can be utilized in ocular delivery of therapeutic proteins and peptides.

## 3. Therapeutic Proteins and Peptides for Ocular Delivery

Size-related challenges faced by the delivery of proteins and peptides through other routes of administration are also experienced in ocular delivery. However, ocular barriers add to these challenges making it more difficult to achieve optimal bioavailability. Proteins and peptides for the treatment of various ocular disorders are discussed below.

Interferon alpha 2b is a topically administered cytokine recommended for the treatment of conjunctiva-cornea intraepithelial neoplasia due to its good side-effect profile and reduction of recurrence. It is both antineoplastic and antiviral via its mechanism of action that suppresses cell proliferation, augments cytotoxic lymphocyte specificity for target cells and hinders viral replication in infected cells [63,64]. Substance P is a neuropeptide found in corneal nerves responsible for corneal wound healing. The neuropeptide is topically administered with other growth factors to stimulate migration, proliferation and differentiation of corneal epithelial cells during corneal injury [65].

Corneal injury treatment includes corneal re-epithelialization which is attained using fibronectin, a glycoprotein existing in plasma as well as extracellular matrices. Fibronectin is also applied topically to treat pertinacious corneal epithelial defects. In the corneal injury, fibronectin collects in the lesion and provides a supplementary matrix for migration and adhesion of epithelial cells, therefore augmenting wound healing and preventing recurrent corneal epithelial defects [66,67].

Another ocular peptide, cyclosporine A (CsA) derived from fungi, is used in the treatment of dry eye syndrome. It is administered as eye drops and can be used for longer periods without showing adverse effects. It also has a reversible and non-toxic action [68]. CsA blocks T-cell activation resulting in inhibition of inflammatory cytokine production. It also inhibits apoptosis via blockade of the mitochondrial permeability transition pore opening and increased conjunctival goblet cell density [69,70].

**Figure 4:**
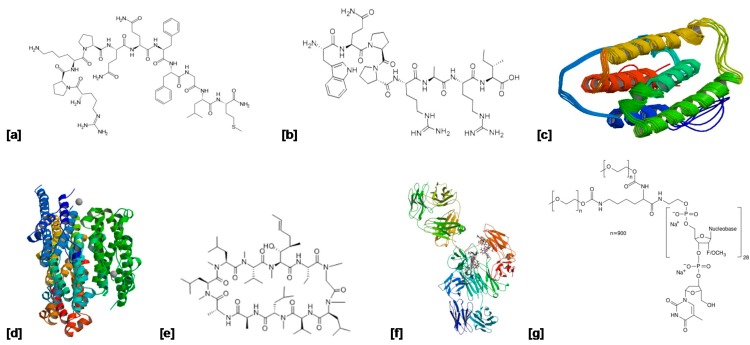
Structures of therapeutic proteins and peptides used in the treatment of ocular/ophthalmic disorders as discussed in Table 2 above. (**a**) Substance P; (**b**) Fibronectin; (**c**)Interferon α2a; (**d**) Interferon α2b; (**e**) Cyclosporine A; (**f**) Bevacizumab; (**g**) Pegaptinib.

The most popular ocular protein therapeutics to date, vascular endothelial growth factor (VEGF) inhibitors, are used in the treatment of chorioretinal diseases such as age-related macular degeneration. These are injected intravitreally to regulate angiogenesis, slow disease progression and eventually reverse the pathogenesis resulting in improved visual acuity and reduced visual loss. Examples include ranibizumab, bevacizumab and pegaptanib. Bevacizumab is preferred for its longer half-life and lower cost compared to ranibizmab [71,72]. Examples of such proteins and peptides are listed in Table 3, as well as ocular disorders for which they are indicated. Structures are shown in Figure 4.

## 4. Current Progress in Stimuli-Responsive Ocular Delivery Systems

### 4.1. In Situ Forming Ophthalmic/Ocular Delivery Systems

In situ-forming systems (Table 4) are the most widely investigated stimuli-responsive systems for ocular delivery of drugs, especially for topical treatment. These are low viscosity, free flowing liquid formulations that undergo sol-gel phase transition when in contact with the stimulus. The transition occurs in the cul-de-sac in topical administration [84]. Their flowability prior to administration puts them at an advantage over systems that are inserted in final form before implantation which require highly invasive surgical procedures for placement into the body.

The rationale for the investigation of in situ-forming ocular/ophthalmic drug delivery systems includes overcoming the following challenges; rapid pre-corneal elimination, normal tear turnover, conjunctival absorption, low bioavailability, frequent administration, high invasion of tissue during implantation, and systemic side-effects due to nasolacrimal drainage of the drug. These lead to insufficient therapeutic levels of the drug and failure to achieve required therapeutic outcome [23,36,38]. A drug dose instilled into the eye is drained at the same time the instillation begins and the dose is eliminated within 5 min. This does not allow enough contact time for therapeutic effect hence the designation of in situ-forming systems [41]. Also, using high concentrations of the drug attempting to overcome the insufficiency of therapeutic drug levels leads to toxicity. Therefore in situ-forming delivery systems have been found to be a solution to this issue. They prolong residence time allowing optimal absorption of the active agent into the tissue and avoid systemic absorption of the drug [85].

Liu and colleagues [89] suggested that ocular therapy would be greatly improved if pre-corneal residence time (drug-tissue contact time) of drugs could be increased. Physiological limitations imposed by protective mechanisms of the eye along with the physiological barriers (tear, cornea, conjunctiva, sclera, choroid, retina and blood retinal barrier) play a role in reducing absorption resulting in short duration of therapeutic effect therefore frequent administration [27]. The amount of drug that penetrates the cornea and reaches intra-ocular tissue is about 1%–6% due to short contact time and drainage [89,90]. This makes it difficult to maintain sufficient amounts of the bioactive in the precorneal area with 75% of the bioactive lost through nasolacrimal drainage and systemic absorption [91,92]. An ideal formulation would be a liquid that will be in contact with the cornea for a longer period of time to improve precorneal residence time and maintain patient compliance [17]. In situ-forming systems have been found to be an alternative that can overcome these limitations, while achieving therapeutic effect without affecting vision and patient acceptability compared to ointments and implants [93].

In situ-forming systems are useful in achieving controlled delivery and minimum invasion of ocular tissue compared to surgical implantation. Derwent and Mieler [39] report on a thermoresponsive hydrogel to deliver VEGF inhibitors to the posterior segment of the eye for the treatment of AMD. Poly (*N*-isopropylacrylamide) (PNIPAAm) was used as the thermoresponsive polymer combined with PEGDA to enhance mechanical strength and prolong release of the peptide. At room temperature was liquid and at 37 °C it solidified within a minute. Thermoresponsive characteristic of the hydrogel makes it possible to inject into the vitreous cavity alternative to intraocular delivery of solid implants which requires tissue invasion [40].

Xie et al. designed an injectable PLGA-PEG-PLGA-based thermoresponsive hydrogel for intravitreal sustained release of Avastin^®^ (bevacizumab) in the treatment of posterior segment disorders. PLGA-PEG-PLGA aqueous solution is injected intravitreally and forms an in situ hydrogel. This study proposes a mechanism of release that entails initial burst release of Avastin^®^ followed by a sustained release with gradual biodegradation of the hydrogel concurrently. In vivo results demonstrated a possibility of an extended Avastin^®^ from the hydrogel in the vitreous humor compared to intravitreal injections [94].

### 4.2. Implantable Stimuli-Responsive Ocular Delivery Systems

Recently, Du Toit and coworkers [95] designed an inflammation-responsive delivery system, called Intelligent Intraocular implant (I^3^), for the treatment of inflammation-related vitreoretinal disorders such as uveitis. I^3^ was designed to respond to inflammation through recognition of a biochemical process specifically immanent to inflammation. Following this recognition is polymeric erosion in the delivery system via an integral mechanism resulting in the release of the incorporated active agent. Hyaluronic acid was employed for its inflammation-responsive properties along with other inflammatory-associated polymers. The system is target specific and biodegradable with minimal side-effects compared to previously studied intraocular implants, owing to its specificity of drug release to the presence of inflammation and therefore improving patient outcomes and optimal drug concentrations at the targeted site.

Li et al. [96] designed a refillable intraocular implant for treatment of anterior and posterior segment diseases. The device is made up of an electrolysis pump, drug reservoir (silicone rubber) and a flexible transcleral cannula with a one-way valve. Silicone rubber membrane can withstand several punctures and reseal without the drug escaping; hence it is a suitable material for the refillable reservoir. Upon application of voltage or current (~50 μA–1.25 mA), electrolysis occurs and consequently the internal pressure increases forcing the valve to open and the drug flows through the cannula into target site; the valve closes upon removal of the stimulus. The amount of drug released is dependent on both the current and duration of exertion. Under normal (5–22 mmHg) and abnormal (>22 mmHg in cases such as glaucoma) intraocular pressure (IOP), the device provided satisfactory results. It is estimated that the device can take up to 24 refills at a 3 month frequency which renders a lifetime of 6 years. Superiority over other ocular implants is the extended lifetime without enlarging the device, refilling without a surgical procedure being required and the fact it permits modification of the drug regimen as per need [97].

Yasin and coworkers [11] reported on a multi-reservoir laser-activated implant, On-demand therapeutics (ODTx), that provides controlled delivery of various active agents to the posterior segment of the eye. Though the patent gives little detail on the composition of ODTx, it reveals that the light responsive part of the implant may consist of a titanium tube or a silicone-based polymer that is extremely impassable. A certain wavelength of electromagnetic radiation focused on a small part of the impassable layer can tear it and release the active agent. ODTx utilizes already existing non-invasive laser technology that is used in treatment of other ocular conditions. One limitation in this implant is that it is non-biodegradable and may result in long-term complications.

Verisome^®^ (Icon Biosciences Inc., Sunnyvale, CA, USA), a biodegradable intravitreal injectable is situ-forming delivery technology that offers long term therapy also reported by Yasin et al. [11] that is undergoing phase II clinical trials for ranibizumab in the treatment of AMD. It is capable of delivering a wide range of molecules (micro and macro) over weeks to a year. The system is injected intravitreally in liquid form and fuses into a spherule that settles posteriorly in the vitreous chamber. The spherule degrades overtime, concurrently releasing the active agent. In preclinical studies, sustained delivery of the active agent in the vitreous was achieved over 6 months and 12 months in 6.9 mg and 13.8 mg formulations, respectively [98,99].

## 5. Future Prospects

The ocular route holds a great potential for opthalmologically-active therapeutic peptides and proteins intended for treatment of ocular diseases. Proteins and peptides considered for local pharmacological action in the eye include polypeptide antibiotics like cyclosporine, tyrothricin, gramicidin, tyrocidine, bacitracin and polymyxins [2]. A number of ophthalmic proteins and peptides in use are listed in Table 3 and majority is commonly administered topically resulting in low bioavailability and systemic side effects, as reviewed. Although these macromolecules are a challenge to administer, especially ocularly due the complex anatomy of the eye and physiological barriers, the stimuli-responsive delivery approach is an alternative to overcome these difficulties.

The use of stimuli-responsive polymers for localized delivery of ocular drugs exists, however it is not popular, particularly with macromolecular proteins and peptides. According to the reviewed literature, in situ gel systems are the most commonly found stimuli-responsive systems in ocular drug delivery, with little done on the delivery of proteins and peptides. Nonetheless, the promising potential of stimuli-responsive polymers can improve ocular delivery of proteins and peptides with minimal invasion of ocular tissue. These polymers can be utilized in protein/peptide delivery to both anterior and posterior segments of the eye to increase site specificity, render sustained delivery, enhance bioavailability and improve patient compliance.

**Figure 5:**
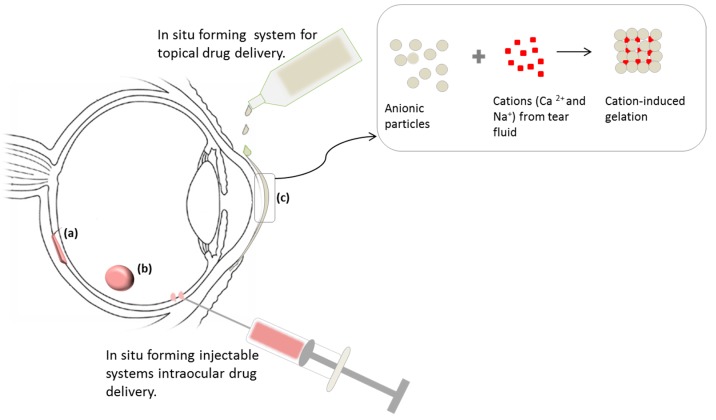
Schematic presentation of possible ocular routes for administration of stimuli responsive drug delivery systems. (**a**) Suprachoroidal implantation, injected as liquid and solidifying in contact with stimuli (e.g., temperature, pH); (**b**) Intravitreal implant which is also injectable and (**c**) Topical eye drops that gel when in contact with the eye due to pH and/or temperature.

Based on the mechanisms in the delivery of proteins and peptides that have been reviewed, we propose that a number of concepts included in Table 2 can be utilized ocularly taking into consideration the stimuli that can be found in the eye and in ophthalmic abnormalities e.g., enzymes (xanthan oxireductase, myeloperoxidase, acid lipase, glutathione-related enzymes, etc.), temperature (~37 °C), inflammation (disease), pH (tear fluid, 7.4), ions and also external stimuli such as light, magnetic field and external thermal sources. With this proposition there exists a prospective achievement of minimally invasive delivery with the main focus in intraocular delivery which is commonly carried out nowadays through surgical implantation and highly invasive intravitreal injections which may lead to ocular complications such as retinal detachment and cataract formation [95]. Also, patient acceptance is likely with minimal invasive administration as an alternative to surgical procedures. In conditions such as age-related macular degeneration, a multi-reservoir stimuli responsive system like the MSN can be used to deliver an anti-vascular endothelial growth factor agent along with an antioxidant and therefore optimizing therapy. It is proposed that antioxidant therapy may be able to minimize disease progress as age-related oxidative changes are part of the causes for AMD [77,100,101]. In Figure 5 we illustrate the probable stimuli and delivery routes for responsive systems to deliver proteins and peptides to both the anterior and posterior segment of the eye.

Responsive delivery of therapeutic proteins and peptides is widely investigated but less work has been done in the ocular route. Therefore our proposition employs concepts from other routes of administration to point to the future of ocular delivery of peptides and proteins. Nonetheless, we take into account challenges that can emerge in the future of this research, such as incompatibilities existing between responsive polymers and the macromolecule of interest and between the stimuli and the ocular tissue. For instance, wavelengths above 900 nm are unable to penetrate ocular tissue [102] and therefore materials that require such wavelengths for activation cannot be employed in ocular delivery. Certain enzyme levels are decreased significantly in some ocular disorders, such as catalase in dry eye disease [103], so systems responsive to such enzymes would be limited.

## 6. Conclusions

This is a futuristic review as it explores the future of stimuli-responsive systems in the delivery therapeutic proteins and peptides via ocular routes. Based on the literature reviewed, challenges may be encountered due to size and instability of the proteins and peptides, however, there is ongoing and advancing research in stimuli-responsive ocular delivery of these macromolecules to both anterior and posterior segments of the eye and it offers great potential. Although only a few stimuli have been investigated in this field to date, it can be deduced from this review that the stimuli possible to employ in ocular delivery can be naturally occurring, such as temperature and pH, disease-specific such as inflammation or external remotely controlled, such as light and magnetic fields. In addition, these responsive systems are a viable solution to the complication of patient compliance due to various delivery approaches that are not patient-friendly.

## Figures and Tables

**Table 1:** A summary of stimuli-responsive polymers introduced to achieve various objectives for the delivery of active agents.

Model Drug	Polymer	Stimuli	Objective	Reference
**Timolol Maleate**	Poloxamer and Chitosan	Temperature	Improved mechanical and mucoadhesive properties and improved retention time for the treatment of ocular diseases.	[18]
**Puerarin**	Poloxamer and Carbopol	Temperature	Maintain sufficient drug concentration at the precorneal area.	[19]
**Pilocarpine Hydrochloride**	Xyloglucan	Temperature	Sustained ocular delivery.	[20,21]
**Ovalbumin**	Poly(*N*-isopropylacrylamide)	Temperature	Prolonged, externally controlled delivery of actives over a specific period of time.	[22]
**Timolol Maleate**	Poly(*N*-isopropylacrylamide) and Chitosan	Temperature	Improved bioavailability and efficacy.	[23]
**Peurarin**	Carbopol and Methylcellulose	Temperature and pH	Improved gel strength, increased precorneal residence time and bioavailability.	[24]
**Ofloxacin**	Polyacrylic acid and HPMC	pH	To provide sustained release of the drug during treatment.	[25]
**Cyclosporine A**	Gellan gum	Electrolytes	Increased drug loading capacity.	[26]
**Gatifloxacin**	Alginate and HPMC	Ions	Enhanced ocular bioavailability and patient compliance.	[27]
**Diclofenac Sodium**	Thiolated poly(aspartic acid)	Oxidation	Prolonged residence time and reduced administration frequency.	[28]

**Table 2:** Stimuli responsive delivery systems for proteins and peptides.

Stimuli	Model Protein/Peptide	Responsive Moiety	Mechanism	Delivery System	Reference
**pH**	Print 3G	Cholesterylhemisuccinate (CHEMS)	CHEMS possesses an inverted cone shape at physiological pH. Under acidic conditions, the carboxylic acid group becomes protonated and CHEMS loses the shape releasing the protein. This transition occurs within endosome where pH is decreased during endocytosis.	Liposomes	[23,36,37]
**pH**	BSA	PLGA-carboxyl group	Undergoes deprotonation at neutral or basic pH which leads to release of the protein.	Micelles	[38,39]
**Enzyme (Human Neutrophil Elastase)**	Anti-inflammatory proteins/peptides	HNE-sensitive peptides (Aminobutyric acid, Novarline, Norleucine)	HNE diffuses into the hydrogel and splits the substrate releasing the therapeutic agent into the site of action.	Hydrogel	[17]
**Enzyme (Chitosanase)**	Fluorescein isothiocyanate-labeled albumin (FITC-albumin)	Chitosan	Enzymatic degradation weakens the capsule wall releasing the protein and the capsule is destroyed over time. Degradation is specific to chitosanase.	Hollow capsules	[40]
**Dual Stimuli (pH, Glucose)**	Insulin	Concanavalin A, *N*-(2-(dimethylamino) ethyl)-methacrylamide	Decrease in pH causes ionization of the amino groups causing swelling. Concanavalin A binds glucose, reducing crosslinking density in mycrohydrogels and increasing hydrophilicity leading to swelling then release of Insulin.	Microhydrogel	[41]

**Table 3:** A summary of the mechanism of action and delivery of ophthalmic proteins and peptides.

Protein/Peptide	Indication	Mechanism of Action	Delivery System	Molecular Weight	Structure (Figure 4)	Reference
**Substance P**	Corneal injury	Stimulates corneal epithelial cell migration and proliferation.	Topical eye drops	~13 kDa	(a)	[73]
**Fibronectin**	Persistent corneal epithelial defect	Functions in corneal re-epithelialization by providing a provisional matrix for epithelial cell adhesion and migration.	Topical eye drops	220 kDa	(b)	[74,75]
**Interferon α2a**	Herpes simplex, keratitis, Macular edema and AMD	Immunomodulatory agent.	Injection (Roferon A^®^)	19 kDa	(c)	[76]
**Interferon α2b**	Ocular surface squamous neoplasia, Conjunctival melanoma	Immunomodulatory agent.	Topical drops and subconjunctival injection (Intron A^®^)	~19 kDa	(d)	[77,78,79]
**Cyclosporine A**	Keratoconjunctivitis sicca and dry eye disease	Immunosuppressive agent that inhibits activation of T lymphocytes.	Eye drops (Restasis^®^)	~12 kDa	(e)	[26,80]
**Bevacizumab**	Age-related macular degeneration	Vascular endothelial growth factor inhibitor. Regulates angiogenesis.	Intravitreal injection (Avastin^®^)	149 kDa	(f)	[81]
**Pegaptinib**	Age-related macular degeneration	Selective antagonist of the 165 isoform of vascular endothelial growth factor–A.	Intravitreal injection (Macugen^®^)	~50 kDa	(g)	[82,83]

**Table 4:** Examples of the in situ-forming ocular drug delivery systems illustrated above.

Bioactive	Polymers	Routes of Administration	Reference
VEGF inhibitors	PNIPAAm	Intravitreal (injection)	[40]
Fluocinolone acetonide	Polyimide	Intravitreal (implant)	[86]
Fuconazole	Gellan gum, carragenan	Topical	[87]
Bevacizumab	Poly (ethylene glycol)-poly (ε-caprolactone)-poly (ethylene glycol)	Intracameral	[88]

## References

[1] Pisal D.S., Kosloski M.P., Balu-Iyer S.V. (2010). Delivery of Therapeutic Proteins. J. Pharm. Sci..

[2] Ratnaparkhi M.P., Chaudhari S.P., Pandya V.A. (2011). Peptides And Proteins In Pharmaceuticals. Int. Curr. Pharm. Res..

[3] Koyamatsu Y., Hirano T., Kakizawa Y., Okano F., Takarada T., Maeda M. (2014). pH-responsive release of proteins from biocompatible and biodegradable reverse polymer micelles. J. Control. Release.

[4] Antosova Z., Mackova M., Kral V., Macek T. (2009). Therapeutic application of peptides and proteins: Parenteral forever?. Trends Biotechnol..

[5] Sharma J.P.K., Bansal S., Banik A. (2011). Noninvasive routes of proteins and peptides drug delivery. Indian J. Pharm. Sci..

[6] Yellepeddi V.K., Palakurthi S. (2016). Recent Advances in Ocular Drug Delivery. Adv. Drug Deliv..

[7] Meshram S., Thorat S. (2015). Ocular in Situ Gels : Development, Evaluation and Advancements. Sch. Acad. J. Pharm..

[8] Vellonen K.S., Malinen M., Mannermaa E., Subrizi A., Toropainen E., Lou Y.R., Kidron H., Yliperttula M., Urtti A. (2014). A critical assessment of in vitro tissue models for ADME and drug delivery. J. Control. Release.

[9] Kaur I.P., Kakkar S. (2014). Nanotherapy for posterior eye diseases. J. Control. Release.

[10] Gaudana R., Jwala J., Boddu S.H.S., Mitra A.K. (2009). Recent perspectives in ocular drug delivery. Pharm. Res..

[11] Yasin M.N., Svirskis D., Seyfoddin A., Rupenthal I.D. (2014). Implants for drug delivery to the posterior segment of the eye: A focus on stimuli-responsive and tunable release systems. J. Control. Release.

[12] Kompella U.B., Amrite A.C., Pacha Ravi R., Durazo S.A. (2013). Nanomedicines for back of the eye drug delivery, gene delivery, and imaging. Prog. Retin. Eye Res..

[13] Kim Y.C., Chiang B., Wu X., Prausnitz M.R. (2014). Ocular delivery of macromolecules. J. Control. Release.

[14] Oak M., Mandke R., Singh J. (2012). Smart polymers for peptide and protein parenteral sustained delivery. Drug Discov. Today Technol..

[15] Roy D., Cambre J.N., Sumerlin B.S. (2010). Future perspectives and recent advances in stimuli-responsive materials. Prog. Polym. Sci..

[16] Ruel-Gariépy E., Leroux J.-C. (2004). In situ-forming hydrogels—review of temperature-sensitive systems. Eur. J. Pharm. Biopharm..

[17] Cohen S., Lobel E., Trevgoda A., Peled Y. (1997). A novel in situ-forming ophthalmic drug delivery system from alginates undergoing gelation in the eye. J. Control. Release.

[18] Gratieri T., Gelfuso G.M., Rocha E.M., Sarmento V.H., de Freitas O., Lopez R.F.V. (2010). A poloxamer/chitosan in situ forming gel with prolonged retention time for ocular delivery. Eur. J. Pharm. Biopharm..

[19] Qi H., Chen W., Huang C., Li L., Chen C., Li W., Wu C. (2007). Development of a poloxamer analogs/carbopol-based in situ gelling and mucoadhesive ophthalmic delivery system for puerarin. Int. J. Pharm..

[20] Miyazaki S., Suzuki S., Kawasaki N., Endo K., Takahashi A. (2001). In situ gelling xyloglucan formulations for sustained release ocular delivery of pilocarpine hydrochloride. Int. J. Pharm..

[21] Brown H.S., Meltzer G., Merrill R.C., Fisher M., Ferré C., Place V.A. (1976). Visual effects of pilocarpine in glaucoma comparative study of administration by eyedrops or by ocular therapeutic systems. Arch. Ophthalmol..

[22] Zhang X.Z., Jo Lewis P., Chu C.C. (2005). Fabrication and characterization of a smart drug delivery system: Microsphere in hydrogel. Biomaterials.

[23] Almeida H., Amaral M.H., Lobao P., Lobo J.M.S. (2014). In situ gelling systems: A strategy to improve the bioavailability of ophthalmic pharmaceutical formulations. Drug Discov. Today.

[24] Wu C., Qi H., Chen W., Huang C., Su C., Li W., Hou S. (2007). Preparation and evaluation of a Carbopol/HPMC-based in situ gelling ophthalmic system for puerarin. Yakugaku Zasshi.

[25] Srividya B., Cardoza R.M., Amin P.D. (2001). Sustained ophthalmic delivery of ofloxacin from a pH triggered in situ gelling system. J. Control. Release.

[26] Gan L., Gan Y., Zhu C., Zhang X., Zhu J. (2009). Novel microemulsion in situ electrolyte-triggered gelling system for ophthalmic delivery of lipophilic cyclosporine A: in vitro and in vivo results. Int. J. Pharm..

[27] Kushwaha S.K., Saxena P., Rai A. (2012). Stimuli sensitive hydrogels for ophthalmic drug delivery: A review. Int. J. Pharm. Investig..

[28] Horvát G., Gyarmati B., Berkó S., Szabó-Révész P., Szilágyi B.Á., Szilágyi A., Soós J., Sandri G., Bonferoni M.C., Rossi S. (2015). Thiolated poly(aspartic acid) as potential in situ gelling, ocular mucoadhesive drug delivery system. Eur. J. Pharm. Sci..

[29] Ye M., Kim S., Park K. (2010). Issues in long-term protein delivery using biodegradable microparticles. J. Control. Release.

[30] Al-tahami K., Singh J. (2007). Smart Polymer Based Delivery Systems for Peptides and Proteins. Recent Pat. Drug Deliv. Formul..

[31] Ducat E., Deprez J., Gillet A., Noël A., Evrard B., Peulen O., Piel G. (2011). Nuclear delivery of a therapeutic peptide by long circulating pH-sensitive liposomes: Benefits over classical vesicles. Int. J. Pharm..

[32] Kim M.S., Lee D.S. (2010). Biodegradable and pH-sensitive polymersome with tuning permeable membrane for drug delivery carrier. Chem. Commun..

[33] Chiu H.C., Lin Y.W., Huang Y.F., Chuang C.K., Chern C.S. (2008). Polymer vesicles containing small vesicles within interior aqueous compartments and pH-responsive transmembrane channels. Angew. Chemie Int. Ed..

[34] El-Sherbiny I.M. (2010). Enhanced pH-responsive carrier system based on alginate and chemically modified carboxymethyl chitosan for oral delivery of protein drugs: Preparation and in-vitro assessment. Carbohydr. Polym..

[35] Gong R., Li C., Zhu S., Zhang Y., Du Y., Jiang J. (2011). A novel pH-sensitive hydrogel based on dual crosslinked alginate/N-α-glutaric acid chitosan for oral delivery of protein. Carbohydr. Polym..

[36] Schmucker C., Loke Y.K., Ehlken C., Agostini H.T., Hansen L.L., Antes G., Lelgemann M. (2011). Intravitreal bevacizumab (Avastin) versus ranibizumab (Lucentis) for the treatment of age-related macular degeneration: A safety review. Br. J. Ophthalmol..

[37] Huth U.S., Schubert R., Peschka-Süss R. (2006). Investigating the uptake and intracellular fate of pH-sensitive liposomes by flow cytometry and spectral bio-imaging. J. Control. Release.

[38] Garbern J.C., Hoffman A.S., Stayton P.S. (2010). Injectable pH- and temperature-responsive poly(N-isopropylacrylamide-co-propylacrylic acid) copolymers for delivery of angiogenic growth factors. Biomacromolecules.

[39] Kang Derwent J.J., Mieler W.F. (2008). Thermoresponsive hydrogels as a new ocular drug delivery platform to the posterior segment of the eye. Trans. Am. Ophthalmol. Soc..

[40] Kumar S., Haglund B.O., Himmelstein K.J. (1994). In Situ -Forming Gels for Ophthalmic Drug Delivery. J. Ocul. Pharmacol. Ther..

[41] Cao Y., Zhang C., Shen W., Cheng Z., Yu L.L., Ping Q. (2007). Poly(*N*-isopropylacrylamide)-chitosan as thermosensitive in situ gel-forming system for ocular drug delivery. J. Control. Release.

[42] Bawa P., Pillay V., Choonara Y.E., du Toit L.C. (2009). Stimuli-responsive polymers and their applications in drug delivery. Biomed. Mater..

[43] Ward M.A., Georgiou T.K. (2011). Thermoresponsive polymers for biomedical applications. Polymers.

[44] Hoffman A.S. (2002). Hydrogels for biomedical applications. Adv. Drug Deliv. Rev..

[45] Ninawe P.R., Hatziavramidis D., Parulekar S.J. (2010). Delivery of drug macromolecules from thermally responsive gel implants to the posterior eye. Chem. Eng. Sci..

[46] Thornton P.D., Mart R.J., Webb S.J., Ulijn R.V. (2008). Enzyme-responsive hydrogel particles for the controlled release of proteins: designing peptide actuators to match payload. Soft Matter.

[47] Aimetti A.A., Tibbitt M.W., Anseth K.S. (2009). Human Neutrophil Elastase Responsive Delivery from Poly( ethylene glycol) Hydrogels. Biomacromolecules.

[48] Itoh Y., Matsusaki M., Kida T., Akashi M. (2006). Enzyme-responsive release of encapsulated proteins from biodegradable hollow capsules. Biomacromolecules.

[49] Yagai S., Kitamura A. (2008). Recent advances in photoresponsive supramolecular self-assemblies. Chem. Soc. Rev..

[50] Shum P., Kim J.M., Thompson D.H. (2001). Phototriggering of liposomal drug delivery systems. Adv. Drug Deliv. Rev..

[51] Peng K., Tomatsu I., Kros A. (2010). Light controlled protein release from a supramolecular hydrogel. Chem. Commun..

[52] Huang Y.F., Sefah K., Bamrungsap S., Chang H.T., Tan W. (2008). Selective photothermal therapy for mixed cancer cells using aptamer-conjugated nanorods. Langmuir.

[53] Kang H., Trondoli A.C., Zhu G., Chen Y., Chang Y.J., Liu H., Huang Y.F., Zhang X., Tan W. (2011). Near-infrared light-responsive core-shell nanogels for targeted drug delivery. ACS Nano.

[54] Jin Q., Cai T., Wang Y., Wang H., Ji J. (2014). Light-Responsive Polyion Complex Micelles with Switchable Surface Charge for Efficient Protein Delivery. ACS Macro Lett..

[55] Zhao H., Sterner E.S., Coughlin E.B., Theato P. (2012). *O*-Nitrobenzyl alcohol derivatives: Opportunities in polymer and materials science. Macromolecules.

[56] Mulvagh S.L., DeMaria A.N., Feinstein S.B., Burns P.N., Kaul S., Miller J.G., Monaghan M., Porter T.R., Shaw L.J., Villanueva F.S. (2000). Contrast echocardiography: current and future applications. J. Am. Soc. Echocardiogr..

[57] Zhao Y.-Z., Du L.-N., Lu C.-T., Jin Y.-G., Ge S.-P. (2013). Potential and problems in ultrasound-responsive drug delivery systems. Int. J. Nanomedicine.

[58] Wang J., Pelletier M., Zhang H., Xia H., Zhao Y. (2009). High-frequency ultrasound-responsive block copolymer micelle. Langmuir.

[59] Suslick K.S. (1990). Sonochemistry. Science.

[60] Casolaro M., Casolaro I., Bottari S., Del Bello B., Maellaro E., Demadis K.D. (2014). Long-term doxorubicin release from multiple stimuli-responsive hydrogels based on α-amino-acid residues. Eur. J. Pharm. Biopharm..

[61] Yin R., Wang K., Du S., Chen L., Nie J., Zhang W. (2014). Design of genipin-crosslinked microgels from concanavalin A and glucosyloxyethyl acrylated chitosan for glucose-responsive insulin delivery. Carbohydr. Polym..

[62] Zakharchenko S., Puretskiy N., Stoychev G., Stamm M., Ionov L. (2010). Temperature controlled encapsulation and release using partially biodegradable thermo-magneto-sensitive self-rolling tubes. Soft Matter.

[63] Scott I.U., Karp C.L., Nuovo G.J. (2002). Human papillomavirus 16 and 18 expression in conjunctival intraepithelial neoplasia. Ophthalmology.

[64] Nemet A.Y., Sharma V., Benger R. (2006). Interferon α2b treatment for residual ocular surface squamous neoplasia unresponsive to excision, cryotherapy and mitomycin-C. Clin. Exp. Ophthalmol..

[65] Nakamura M., Kawahara M., Nakata K., Nishida T. (2003). Restoration of corneal epithelial barrier function and wound healing by substance P and IGF-1 in rats with capsaicin-induced neurotrophic keratopathy. Investig. Ophthalmol. Vis. Sci..

[66] Nishida T. (2012). The role of fibronectin in corneal wound healing explored by a physician-scientist. Jpn. J. Ophthalmol..

[67] Cameron J.D., Skubitz A.P., Furcht L.T. (1991). Type IV collagen and corneal epithelial adhesion and migration. Effects of type IV collagen fragments and synthetic peptides on rabbit corneal epithelial cell adhesion and migration in vitro. Invest. Ophthalmol. Vis. Sci..

[68] Kymionis G.D., Bouzoukis D.I., Diakonis V.F., Siganos C. (2008). Treatment of chronic dry eye: Focus on cyclosporine. Clin. Ophthalmol..

[69] Kunert K.S., Tisdale A.S., Gipson I.K. (2002). Goblet cell numbers and epithelial proliferation in the conjunctiva of patients with dry eye syndrome treated with cyclosporine. Arch Ophthalmol..

[70] Matsuda S., Koyasu S. (2000). Mechanisms of action of cyclosporine. Immunopharmacology.

[71] Bakri S.J., Snyder M.R., Reid J.M., Pulido J.S., Ezzat M.K., Singh R.J. (2007). Pharmacokinetics of Intravitreal Ranibizumab (Lucentis). Ophthalmology.

[72] Agrahari V., Agrahari V., Hung W.-T., Christenson L., Mitra A.K. (2016). Composite Nanoformulation Therapeutics for Long Term Ocular Delivery of Macromolecules. Mol. Pharm..

[73] Kingsley R.E., Marfurt C.F. (1997). Topical substance P and corneal epithelial wound closure in the rabbit. Invest. Ophthalmol. Vis. Sci..

[74] Nelson J.D., Gordon J.F. (1992). Topical fibronectin in the treatment of keratoconjunctivitis sicca. Chiron Keratoconjunctivitis Sicca Study Group. Am. J. Ophthalmol..

[75] McCulley J.P., Horowitz B., Husseini Z.M., Horowitz M. (1993). Topical fibronectin therapy of persistent corneal epithelial defects. Fibronectin Study Group. Trans. Am. Ophthalmol. Soc..

[76] Finger P.T., Sedeek R.W., Chin K.J. (2008). Topical Interferon Alfa in the Treatment of Conjunctival Melanoma and Primary Acquired Melanosis Complex. Am. J. Ophthalmol..

[77] Jarrett S.G., Boulton M.E. (2012). Consequences of oxidative stress in age-related macular degeneration. Mol. Aspects Med..

[78] Gantyala S.P., Shekhar H., Vanathi M., Sinha R., Titiyal J.S. (2013). Interferons in Ophthalmology Current Status and Advancing Trends. Delhi J. Ophthalmol..

[79] Huerva V., Mangues I. (2008). Treatment of conjunctival squamous neoplasias with interferon alpha 2ab. J. Fr. Ophtalmol..

[80] Shen J., Deng Y., Jin X., Ping Q., Su Z., Li L. (2010). Thiolated nanostructured lipid carriers as a potential ocular drug delivery system for cyclosporine A: Improving in vivo ocular distribution. Int. J. Pharm..

[81] Rauck B.M., Friberg T.R., Medina Mendez C.A., Park D., Shah V., Bilonick R.A., Wang Y. (2013). Biocompatible reverse thermal gel sustains the release of intravitreal bevacizumab in vivo. Investig. Ophthalmol. Vis. Sci..

[82] Yin R., Tong Z., Yang D., Nie J. (2012). Glucose-responsive insulin delivery microhydrogels from methacrylated dextran/concanavalin A: preparation and in vitro release study. Carbohydr. Polym..

[83] Velez-montoya R., Oliver S.C.N., Olson J.L., Fine S.L., Mandava N., Quiroz-mercado H. (2013). Age-Related Macular Degeneration: Today’s and Future Treatments. J. Ret. Vit. Dis..

[84] Kute R.B., Gondkar P.R., Saudagar S.B. (2015). Ophthalmic In-situ Gel: An Overview. World J. Pharm. Pharm. Sci..

[85] Sieg J.W., Robinson J.R. (1981). Mechanistic studies on transcorneal permeation of fluorometholone. J. Pharm. Sci..

[86] Zarbin M.A., Rosenfeld P.J. (2010). Pathway-based therapies for age-related macular degeneration: an integrated survey of emerging treatment alternatives. Retina.

[87] Athanasakis K., Fragoulakis V., Tsiantou V., Masaoutis P., Maniadakis N., Kyriopoulos J. (2012). Cost-effectiveness analysis of ranibizumab versus verteporfin photodynamic therapy, pegaptanib sodium, and best supportive care for the treatment of age-related macular degeneration in Greece. Clin. Ther..

[88] Peng R., Qin G., Li X., Lv H., Qian Z., Yu L. (2014). The PEG-PCL-PEG Hydrogel as an Implanted Ophthalmic Delivery System after Glaucoma Filtration Surgery; a Pilot Study. Med. hypothesis, Discov. Innov. Ophthalmol..

[89] Liu Z., Li J., Nie S., Liu H., Ding P., Pan W. (2006). Study of an alginate/HPMC-based in situ gelling ophthalmic delivery system for gatifloxacin. Int. J. Pharm..

[90] Shastri D., Patel L., Parikh R. (2010). Studies on In situ Hydrogel: A Smart Way for Safe and Sustained Ocular Drug Delivery. J. Young Pharm..

[91] Maurice D.M., Mishima S. (1984). Ocular Pharmacokinetics.

[92] Kuno N., Fujii S. (2011). Recent Advances in Ocular Drug Delivery Systems. Polymers.

[93] Schoenwald R. (1997). Textbook of Ocular Pharmacology.

[94] Xie B., Jin L., Luo Z., Yu J., Shi S., Zhang Z., Shen M., Chen H., Li X., Song Z. (2015). An injectable thermosensitive polymeric hydrogel for sustained release of Avastin1 to treat posterior segment disease. Int. J. Pharm..

[95] Du Toit L.C., Carmichael T., Govender T., Kumar P., Choonara Y.E., Pillay V. (2014). In vitro, in vivo, and in silico evaluation of the bioresponsive behavior of an intelligent intraocular implant. Pharm. Res..

[96] Li P., Shih J., Lo R., Saati S., Agrawal R., Humayun M.S., Tai Y., Meng E. (2008). An electrochemical intraocular drug delivery device. Sens. Actuators.

[97] Lo R., Li P., Saati S., Agrawal R., Humayun S., Meng E. (2008). A refillable microfabricated drug delivery device for treatment of ocular diseases. Lab Chip.

[98] Haghjou N., Soheilian M., Abdekhodaie M.J. (2011). Sustained release intraocular drug delivery devices for treatment of uveitis. J. Ophthalmic Vis. Res..

[99] Parlato M., Johnson A., Hudalla G.A., Murphy W.L. (2013). Adaptable poly(ethylene glycol) microspheres capable of mixed-mode degradation. Acta Biomater..

[100] Oubraham H., Cohen S.Y., Samimi S., Marotte D., Bouzaher I., Bonicel P., Fajnkuchen F., Tadayoni R. (2011). Inject and extend dosing versus dosing as needed: A comparative retrospective study of ranibizumab in exudative age-related macular degeneration. Retina.

[101] Prasad P.S., Schwartz S.D., Hubschman J.-P. (2010). Age-related macular degeneration: Current and novel therapies. Maturitas.

[102] Juzenas P., Juzeniene A., Kaalhus O., Iani V., Moan J. (2002). Noninvasive fluorescence excitation spectroscopy during application of 5-aminolevulinic acid in vivo. Photochem. Photobiol. Sci..

[103] Malec J., Dot D., Cejková J., Br B. (2008). Decreased expression of antioxidant enzymes in the conjunctival epithelium of dry eye (Sjögren’s syndrome) and its possible contribution to the development of ocular surface oxidative injuries. Histol. Histopathol..

